# Case Report: combined surgical and pharmacological management of basal ganglia cryptococcal meningoencephalitis

**DOI:** 10.3389/fmed.2025.1601269

**Published:** 2025-08-06

**Authors:** Xiao-Yu Zheng, Ping Chen, Dong-Yue Li, Ke-En Chen, Jian-Cheng Liao, Meng-Hui Li, Mao-Ying Zhang

**Affiliations:** ^1^Department of Intensive-Care Unit, The First Affiliated Hospital of Jinan University, Guangzhou, China; ^2^Department of Neurosurgery, The First Affiliated Hospital of Jinan University, Guangzhou, China

**Keywords:** cryptococcal meningoencephalitis, basal ganglia, combined therapy, case report, neurosurgical intervention

## Abstract

**Introduction:**

Cryptococcal meningoencephalitis involving the basal ganglia is exceedingly rare and poses diagnostic and therapeutic challenges due to its tumor-mimicking presentation. This case underscores the necessity of a multidisciplinary approach integrating surgical intervention and targeted antifungal therapy.

**Case presentation:**

A 47-years-old immunocompetent female presented with progressive headache and left-sided hemiparesis. Neuroimaging identified a basal ganglia mass initially misdiagnosed as a neoplastic lesion.

**Interventions:**

Surgical resection under neuroelectrophysiological monitoring was combined with amphotericin B, flucytosine, and fluconazole.

**Outcomes:**

Complete lesion excision, symptom resolution, and mild memory deficits at 4-years follow-up.

**Lessons:**

Combined therapy is pivotal for CM with parenchymal involvement; early surgery may mitigate irreversible neurological sequelae.

## Introduction

Cryptococcal meningoencephalitis predominantly affects immunocompromised individuals, with parenchymal granulomas in the basal ganglia being exceedingly rare (< 1% of cases). Current guidelines emphasize pharmacological therapy; however, surgical management remains controversial for lesions in functional brain regions. This report delineates the first documented case of basal ganglia CM managed via combined craniotomy and triple antifungal therapy, challenging conventional reliance on conservative strategies.

### Case description

**FIGURE 1:**
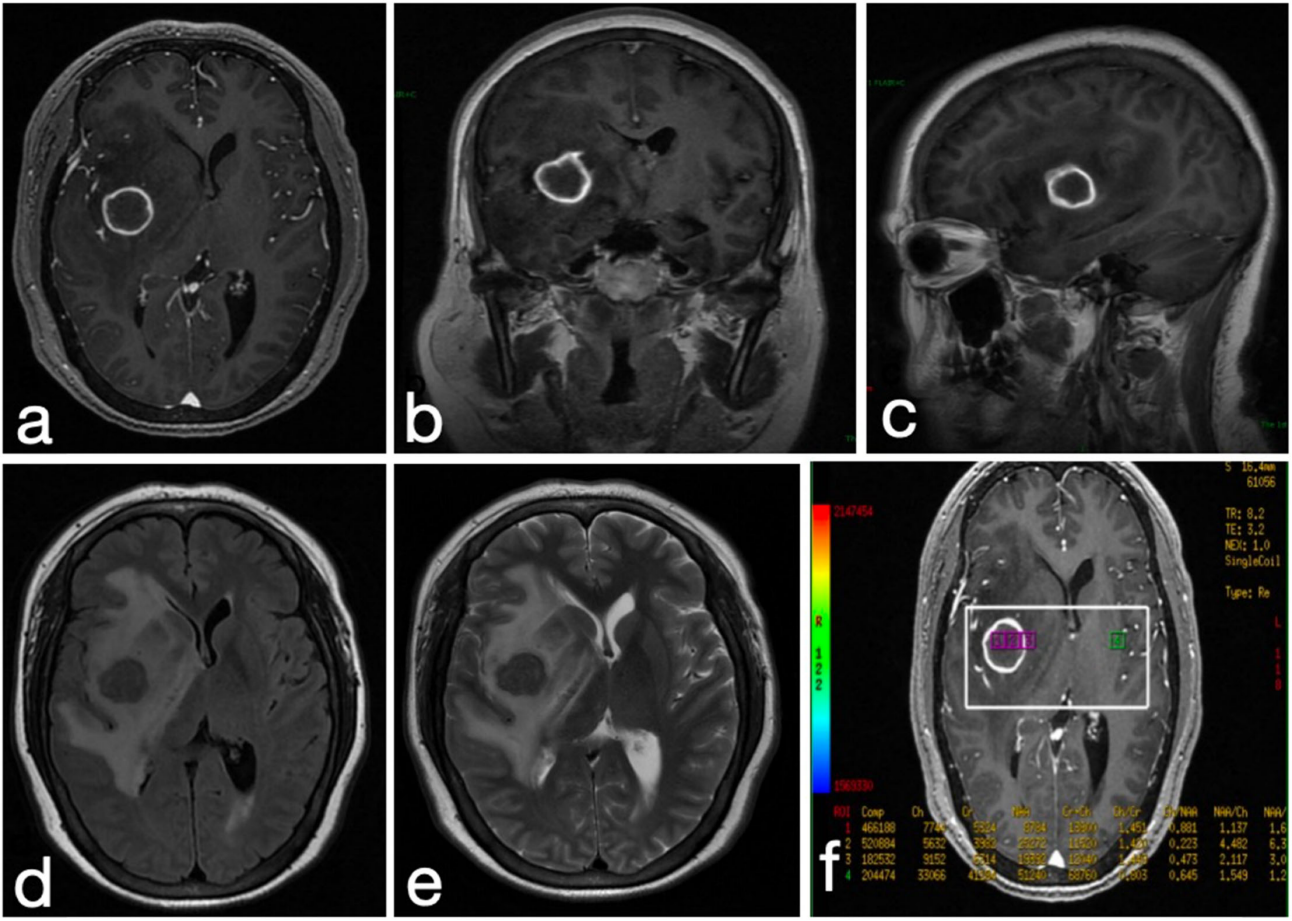
Imaging revealed a mass in the right basal ganglia with extensive perifocal edema **(a)** T1 enhanced axial, **(b)** T1 enhanced coronal, **(c)** T1 enhanced sagittal, **(d)** T2 Flair axial, **(e)** T2 axial, **(f)** MR spectra.

**FIGURE 2:**
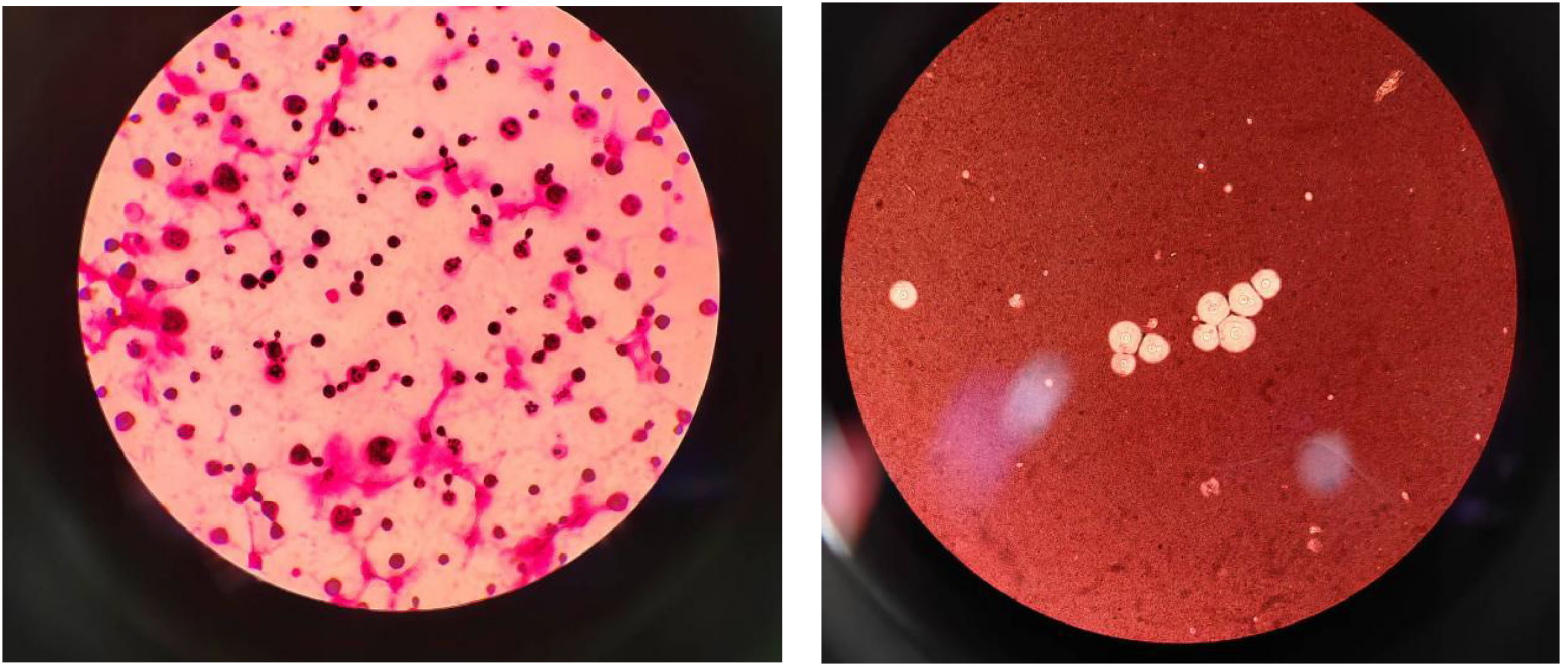
The left picture shows cryptococcal Gram stain; the right picture shows India ink stainings was positive.

A 47-years-old immunocompetent female with no history of HIV infection or immunosuppression presented with a 1-year history of progressive headache and acute-onset left-sided limb weakness evolving over a 4-days period. Importantly, the patient worked as a pigeon breeder, which likely contributed to her exposure to Cryptococcus via pigeon droppings. Neurological examination demonstrated left hemiparesis (muscle strength grade IV/V) and meningeal irritation signs, without concurrent fever or papilledema. Initial MRI revealed a 2.5 cm ring-enhancing lesion in the right basal ganglia with perilesional edema (Figures 1a–f), suggestive of a neoplasm or tuberculoma. Cerebrospinal fluid (CSF) analysis confirmed cryptococcal meningoencephalitis [India ink stain positive (Figure 2); cryptococcal antigen lateral flow assay (LFA) positive], with an elevated opening pressure of 280 mmH2O. On postoperative day 5, craniotomy with intraoperative neuroelectrophysiological monitoring achieved complete lesion resection. Postoperatively, she received a 3-weeks induction regimen of amphotericin B (0.7 mg/kg/d), flucytosine (100 mg/kg/d), and fluconazole (800 mg/d), followed by 6 months of fluconazole consolidation therapy. Follow-up MRI confirmed lesion excision, and the CSF cryptococcal antigen test became negative 1 month after initiation of therapy. During the diagnostic process, the pathogen was identified as Cryptococcus neoformans via fungal culture and molecular biological identification.

**FIGURE 3:**
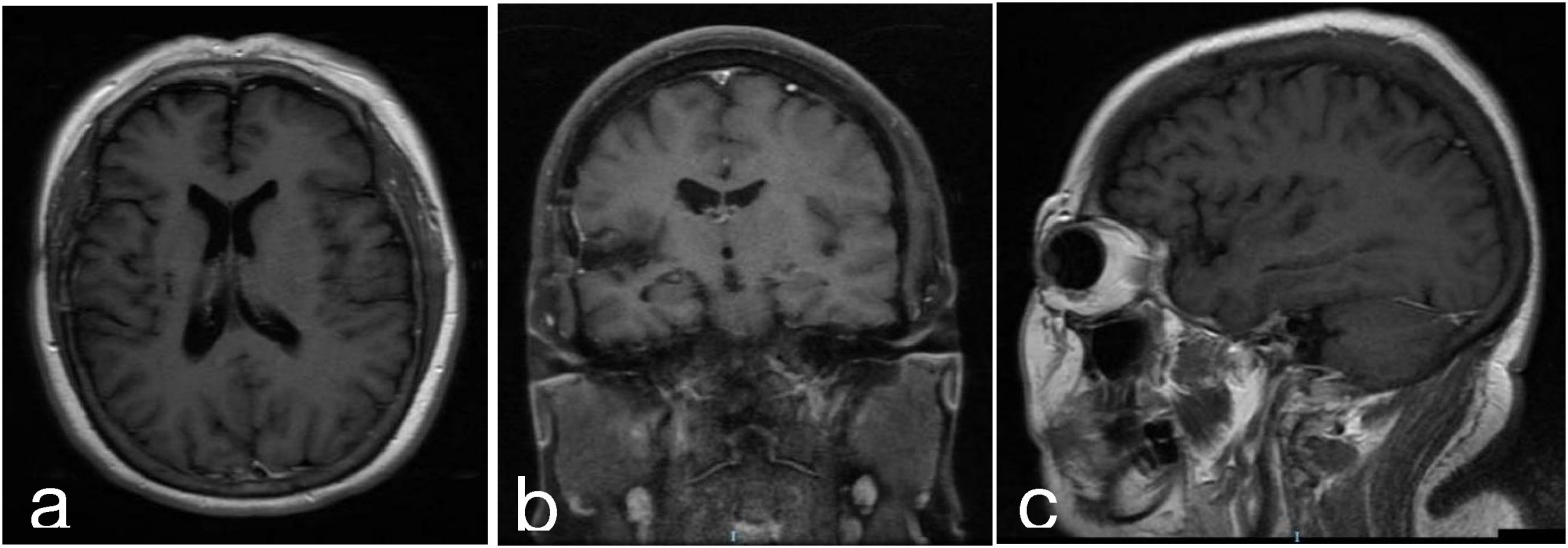
Postoperative MRI of the same patient in Figure 1 after 4 years **(a)** T1 enhanced axial, **(b)** T1 enhanced coronal, **(c)** T1 enhanced sagittal.

At the 4-years follow-up, the patient reported mild episodic memory impairment but exhibited no motor deficits or radiological recurrence (Figures 3a–c). Table 1 showcasing a timeline with relevant data from the episode of care.

**TABLE 1:** Timeline of care.

Date	Event	Outcome
Day 1	Admission; MRI showing right basal ganglia lesion	Suspected tumor/abscess
Day 3	CSF analysis: positive India ink stain; cryptococcal antigen lateral flow assay (LFA) positive	Confirmed CM
Day 5	Craniotomy with complete lesion resection	No intraoperative complications
Post-operation	Amphotericin B + flucytosine + fluconazole × 3 weeks	Symptom resolution
4-years follow-up	MRl and neuropsychological testing	No recurrence; mild memory deficits

## Discussion

This case of basal ganglia cryptococcal meningoencephalitis (CM) in an immunocompetent patient illustrates the diagnostic and therapeutic challenges posed by parenchymal cryptococcal lesions, particularly in immunocompetent populations. Emerging evidence highlights an increased incidence of cryptococcal infections in immunocompetent individuals, potentially attributable to environmental exposures or subclinical immune dysregulation (1). Our findings corroborate emerging reports advocating multidisciplinary strategies for complex CM cases while identifying critical gaps in current guidelines (2).

### Strengths and innovations

The integration of surgical resection with triple antifungal therapy (amphotericin B, flucytosine, fluconazole) constitutes a paradigm shift in the management of parenchymal CM. While the 2022 IDSA guidelines prioritize medical management (3), recent studies advocate early surgical intervention for lesions causing mass effect or refractory intracranial hypertension. A study by Iyer KR et al. (4) demonstrated that surgical debulking combined with antifungals reduced mortality by 40% in non-HIV CM patients with parenchymal involvement, validating our approach. Furthermore, intraoperative neuroelectrophysiological monitoring–employed here to preserve functional integrity–has garnered increasing recognition in recent years. A 2023 systematic review underscored its role in minimizing postoperative deficits in deep brain lesions (5), reinforcing our technical strategy.

The selection of antifungal agents aligns with contemporary therapeutic trends. Single-dose liposomal amphotericin B combined with flucytosine and fluconazole was non-inferior to the WHO-recommended treatment for HIV-associated cryptococcal meningitis and was associated with fewer adverse events (6). While fluconazole was employed for consolidation in this case, future cases could potentially benefit from these advances to mitigate relapse risks.

### Limitations and unresolved questions

Despite favorable outcomes, this approach is not without limitations. First, risks inherent to basal ganglia surgery–including hemorrhage or cognitive impairment–persist as significant concerns. The patient’s mild memory impairment is consistent with a 2022 cohort study reporting subtle neurocognitive deficits in 30% of survivors after ventriculoperitoneal shunt placement (7). Second, the optimal duration of antifungal therapy post-surgical resection remains uncertain. Current guidelines recommend 6–12 months of fluconazole (3); however, a 2023 meta-analysis suggested extending this to 18 months for parenchymal cases (8), a strategy necessitating further validation.

### Comparison with recent literature

Recent literature has increasingly recognized the heterogeneous clinical presentations of CM. A case report by Kelly et al. (9) reported three immunocompetent patients with basal ganglia cryptococcomas, all initially misdiagnosed as neoplastic lesions. Similar to our case, surgical resection facilitated definitive diagnosis and symptom resolution, highlighting the critical role of histopathology in atypical presentations. However, their patients received shorter antifungal courses (3–6 months), with one case experiencing relapse at 2 years–a risk potentially mitigated by our prolonged regimen.

The role of novel diagnostics is another evolving area. While CSF cryptococcal antigen testing remains pivotal, a 2023 study demonstrated the utility of metagenomic next-generation sequencing (mNGS) in rapidly identifying cryptococcal DNA in brain biopsies (10). Adoption of such technologies could expedite diagnostic workflows in future cases.

### Clinical implications and future directions

Key lessons from this case include: (1) Early surgical biopsy should be considered for lesions in functional brain regions, even in immunocompetent patients, to prevent diagnostic delays. (2) Personalized antifungal regimens–tailored to lesion burden and immune status–could enhance clinical outcomes. Emerging therapies, including lipid-based amphotericin formulations (11) or adjunctive immunomodulators [e.g., interferon-γ (12)], warrant further investigation. (3) Long-term neurocognitive monitoring is imperative, as subtle deficits may persist despite radiographic resolution.

## Conclusion

This case challenges the traditional reliance on conservative therapy for parenchymal CM and advocates individualized, risk-stratified management strategies. Future studies ought to investigate standardized protocols for surgical candidacy and antifungal duration in comparable clinical scenarios.

## Data Availability

The original contributions presented in this study are included in this article/supplementary material, further inquiries can be directed to the corresponding authors.
